# Profiling and integrated analysis of transcriptional addiction gene expression and prognostic value in hepatocellular carcinoma

**DOI:** 10.18632/aging.204676

**Published:** 2023-04-22

**Authors:** Xiaowei Du, Hao Wang, Jing Xu, Yufei Zhang, Tingsong Chen, Gao Li

**Affiliations:** 1 First Affiliated Hospital of Fujian Medical University, Fuzhou, China; 2 Second Department of Oncology, Seventh People’s Hospital of Shanghai University of Traditional Chinese Medicine, Shanghai, China

**Keywords:** transcriptional addiction, nomogram, prognostic signature, hepatocellular carcinoma, TCGA

## Abstract

Transcriptional dysregulation caused by genomic and epigenetic alterations in cancer is called “transcriptional addiction”. Transcriptional addiction is an important pathogenic factor of tumor malignancy. Hepatocellular carcinoma (HCC) genomes are highly heterogeneous, with many dysregulated genes. Our study analyzed the possibility that transcriptional addiction-related genes play a significant role in HCC. All data sources for conducting this study were public cancer databases and tissue microarrays. We identified 38 transcriptional addiction genes, and most were differentially expressed genes. Among patients of different groups, there were significant differences in overall survival rates. Both nomogram and risk score were independent predictors of HCC outcomes. Transcriptional addiction gene expression characteristics determine the sensitivity of patients to immunotherapy, cisplatin, and sorafenib. Besides, HDAC2 was identified as an oncogene, and its expression was correlated with patient survival time. Our study conclusively demonstrated that transcriptional addiction is crucial in HCC. We provided biomarkers for predicting the prognosis of HCC patients, which can more precisely guide the patient’s treatment.

## INTRODUCTION

Primary liver cancer is the sixth most commonly diagnosed cancer worldwide [1]. China is a high-risk area for liver cancer, with the second-highest incidence rate among all malignant cancers [2]. Hepatocellular carcinoma (HCC) accounts for 75-85% of all liver cancers. There are several drug strategies for HCC, including chemotherapy, targeted therapy, and immunotherapy, but the drug response varies significantly among cancer patients [3, 4]. Considering the tumor heterogeneity and multiple risk factors, biomarkers for HCC should be individualized and diversified.

The dysregulation of transcription is crucial for tumorigenesis and development [5]. Transcriptional dysregulation caused by genomic and epigenetic alterations is called “transcriptional addiction” [6]. Transcriptional addiction in cancer cells can be overly dependent on various components of the transcriptional process [7]. Therefore, small molecule inhibitors targeting transcriptional processes may be more effective against these cancer cells [5]. Targeting transcriptional cyclin-dependent kinases and small molecule proteins is a promising treatment strategy that could contribute to the rational design of cancer combination therapies.

The oncogenic effect of transcriptional addiction has been demonstrated in several cancers. The efficacy of CDK7 inhibitors depends on the selective dependency of tumors on CDK7 kinase activity. For instance, CDK7 inhibitors could selectively target tumor cells and suppress the growth of breast tumors [7]. The repression of CDK7 may promote marked transcriptional dysregulation in ovarian cancer, which is not recognized as an actionable vulnerability [8]. Identifying transcriptional addiction genes may aid in developing HCC biomarkers and therapeutic strategies.

The present study systematically analyzed the expression levels and prognostic value of transcriptional addiction genes in HCC. Clustered subgroups and risk signatures provided predictable biomarkers of patients’ survival. Among these genes, HDAC2—an oncogene—was associated with patient survival. These findings substantiate the great potential of transcriptional addiction in HCC.

## RESULTS

### Identification of differentially expressed genes

We identified a series of genes associated with cancer transcriptional addiction (n=38) through a focused literature review [6–39] (Table 1). We refer to these genes as transcriptional addiction genes.

**Table 1:** Literature review identified the transcriptional addiction genes.

**Cancer type**	**Gene list**
Acute myelocytic leukemia [6, 9-15]	DOT1L, BRD4, MEF2D, IRF8, KMT2A, ZMYND8, MEF2D, CDK9, ZFP64
T cell leukemia [6]	CDK7, BRD4
Multiple myeloma [6]	BRD4
Glioma [6, 16]	PDGFRA, CDK7
Melanoma [7]	YAP, TAZ
Neuroblastoma [17-19]	TBX2, FOXM1, CDK7, CDK12,
Liposarcoma [20]	FOSL2, MYC, RUNX1, SNAI2
Bone and soft tissue sarcomas [21]	CDK7
Gastrointestinal Stromal Tumor [22]	FOXF1, ETV1
Thyroid cancer [23-25]	RUNX2, FOXC1, CDK7, PPP1R15A
Esophageal cancer [19, 26]	HDAC1, HDAC2, TP63, SOX2, KLF5
Breast cancer [7, 18, 27-30]	YAP, TAZ, BRD4, Skp2, EN1, TRPS1, CDK7, CDK12
lung cancer [18, 31-33]	CDK7, SOX2, MYC, CDK12
Liver cancer [7]	YAP, TAZ, SOX9
Gallbladder Cancer [34]	CDK7
Pancreatic cancer [35]	CDK7
Colon cancer [36]	P53, CDK7
Bladder cancer [18, 37]	CDK7, CDK12
Ovarian Cancer [8]	CDK7
Prostate cancer [38, 39]	CDK7, CDK9, CDK8, CDK19, CDK12, CDK13, MED1

**Figure 1:**
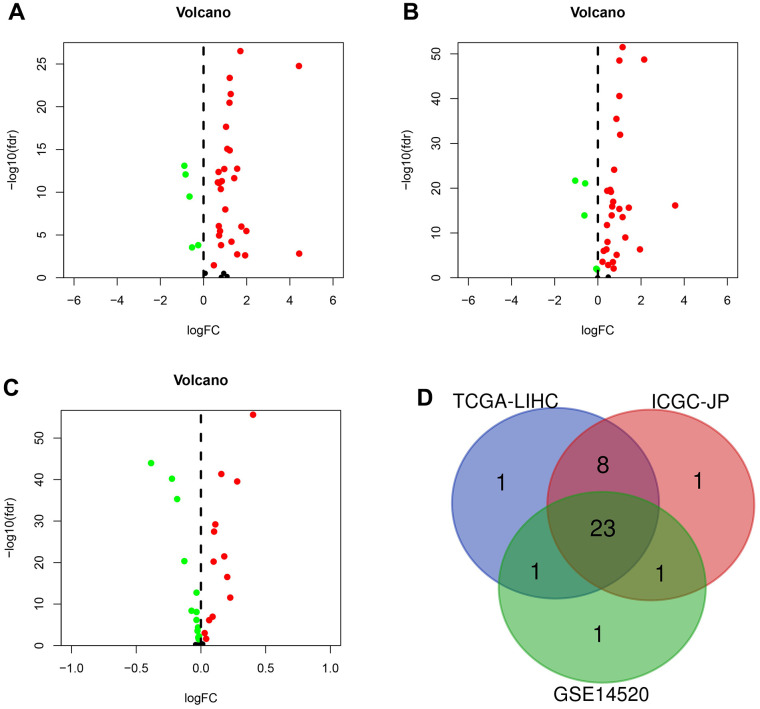
**The differentially expressed genes (DEGs) were screened in different sets.** (**A**) DEGs (n = 33) in TCGA-LIHC set. (**B**) DEGs (n = 33) in the ICGC-JP set. (**C**) DEGs (n = 26) in GEO-GSE14520 set. (**D**) Venn diagram for the overlapping genes.

We identified DEGs in HCC from three platforms. In the TCGA-LIHC set, 33 genes (86.8%) were differentially expressed in tumor tissues and paraneoplastic tissues (*p* < 0.05) (Figure 1A). We identified 33 and 26 DEGs in the ICGC-JP set and GEO-GSE14520 set, respectively (Figure 1B, 1C). The analysis results were consistent across the three platforms, with most genes overlapping (Figure 1D).

### Transcription factor co-expression network and gene function analysis

**Figure 2:**
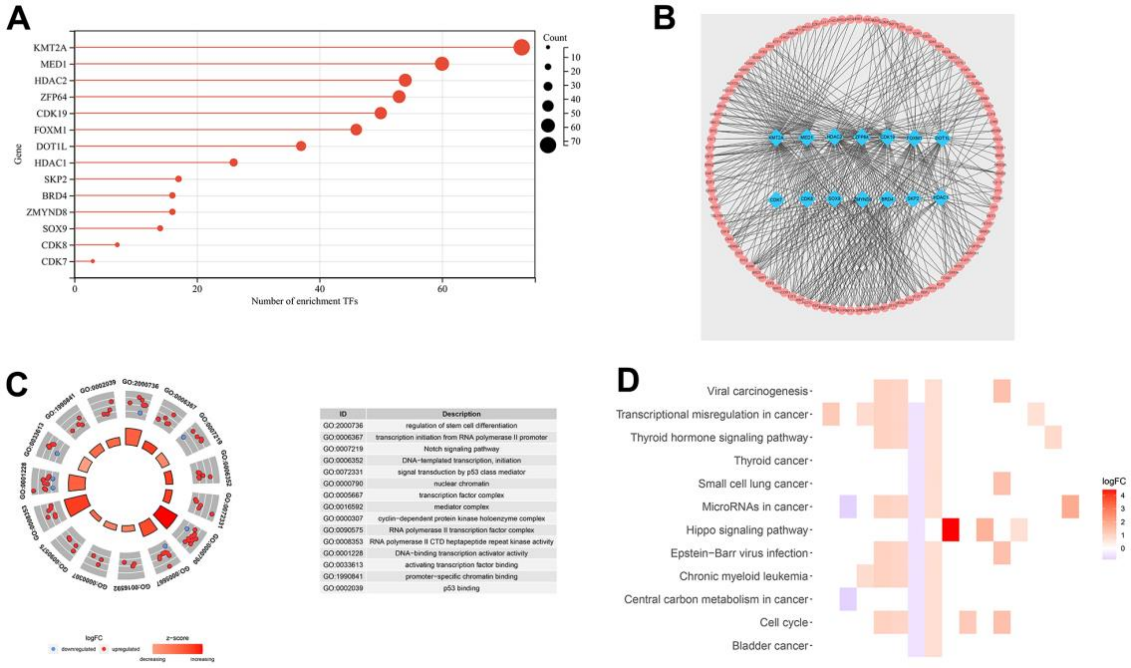
**Transcription factor co-expression network and gene function analysis for TCGA-LIHC set.** (**A**) The bar chart shows the number of transcription factors related to DEGs. (**B**) Co-expression network of 14 DEGs and 314 transcription factors. (**C**) Functional annotation for transcriptional addiction genes using GO term enrichment analysis, according to the top five biological processes, the top five cellular components, and the top five molecular functions. (**D**) The top 15 KEGG enrichment pathways of transcriptional addiction genes.

There was a strong correlation (|R^2^| > 0.5 and *p* < 0.05) between the expression of 14 DEGs and tumor transcription factors (Figure 2A). The top three DEGs were KMT2A, MED1, and HDAC2. The expression of these 14 DEGs in cancer tissues was higher than that in paracancerous tissues. There were 314 transcription factors screened using the correlation analysis. We plotted the co-expression network using the Cytoscape v3.8.1 software (Figure 2B).

Functional enrichment analysis was performed based on 33 DEGs from the TCGA-LIHC set. The GO dataset enriched the regulation of stem cell differentiation, transcription initiation from RNA polymerase II promoter, and DNA−templated transcription (Figure 2C). As for the KEGG dataset, viral carcinogenesis, transcriptional misregulation in cancer, and microRNAs in cancer were enriched (Figure 2D). Transcription-related functions were enriched in both datasets.

### Clustered subgroups and nomograms for predicting patient survival in the TCGA-LIHC set

**Figure 3:**
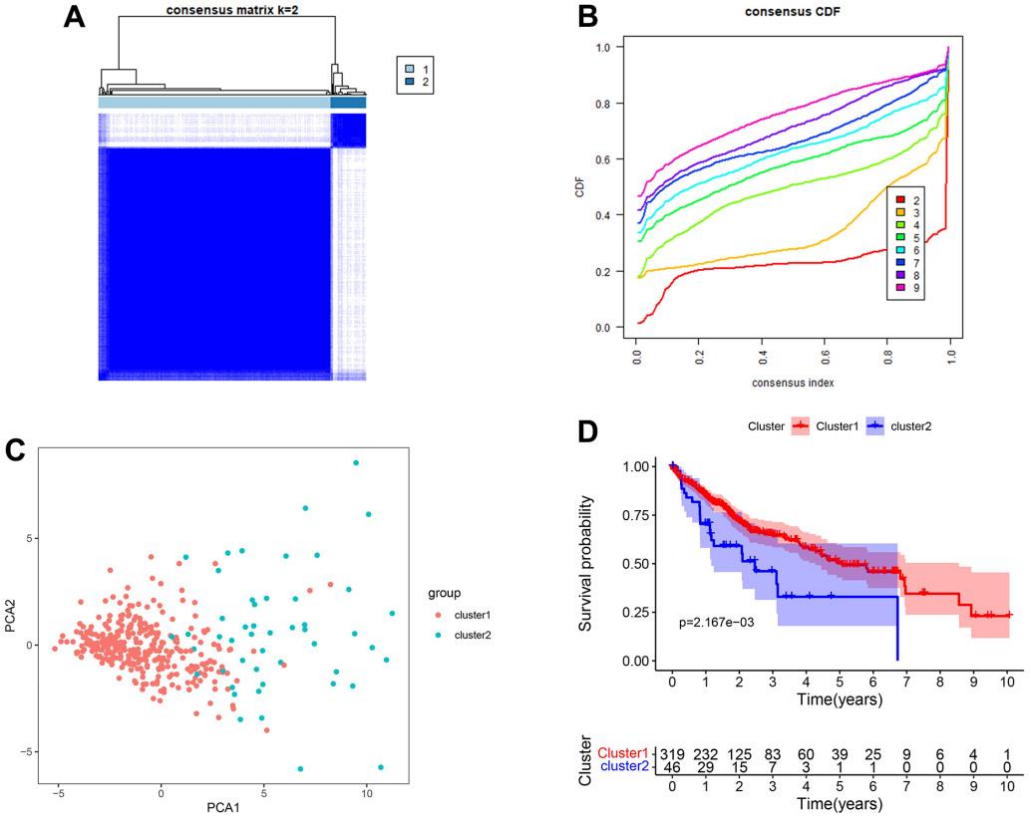
**Clustered subgroups of DEGs in TCGA-LIHC set.** (**A**, **B**) K = 2 was chosen to construct the clustered subgroups. (**C**) Principal component analysis of transcriptional addiction genes to distinguish different clustered subgroups. (**D**) Survival analysis for the gene clusters based on 365 patients from TCGA-LIHC set.

For the best clustering effect, we chose k = 2 for the subsequent analyses (Figure 3A, 3B). The different clustering subgroups revealed a two-way distribution through PCA analyses (Figure 3C). According to the survival analysis, the patients in subgroup 1 exhibited higher one-, three-, and five-year overall survival rates than those in subgroup 2 (log-rank test *p* < 0.01) (Figure 3D).

**Figure 4:**
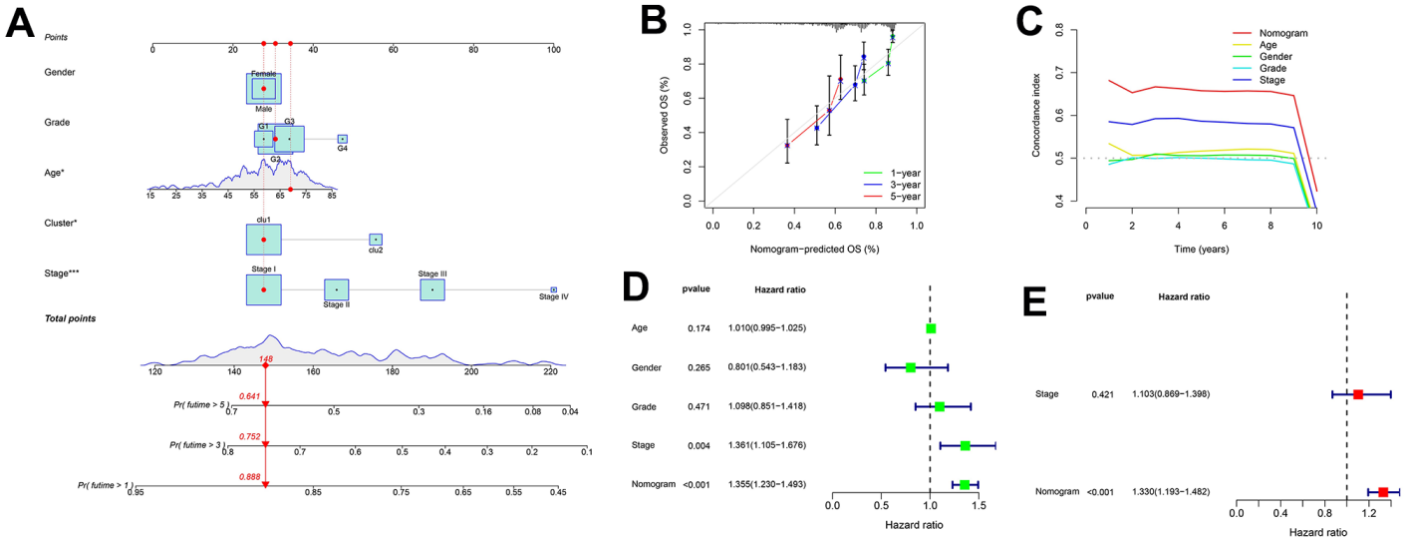
**Nomogram for predicting patient survival in TCGA-LIHC set.** (**A**) The nomogram combines the cluster and clinicopathological characteristics for predicting patient survival outcomes. (**B**) The calibration curve validates the sensitivity of the nomogram in 1, 3, and 5 years. (**C**) The C-index validates the specificity of the nomogram compared to the clinicopathological characteristics. (**D**, **E**) Univariate and multivariate regression analyses for the nomogram as an independent prognostic factor.

The independent variables used to construct the nomogram include age, gender, grade, stage, and cluster (Figure 4A). The calibration curve and C-index confirm the high predictive accuracy and specificity of the nomogram (Figure 4B, 4C). In univariate/multivariate Cox regression analyses, the nomogram was an independent predictor of HCC outcome (HR > 1 and *p* < 0.001) (Figure 4D, 4E).

### Identification of an 11-gene risk signature for predicting patient survival

The 11-gene risk signature was constructed using the training set (Table 2). Each patient’s risk score can be calculated using the following formula. Risk score = (-0.908 * expression value of EN1) + (0.214 * expression value of ETV1) + (0.238 * expression value of HDAC2) + (-0.058 * expression value of IRF8) + (0.017 * expression value of MYC) + (0.020 * expression value of SNAI2) + (0.030 * expression value of TAZ) + (-0.073 * expression value of TP53) + (-0.218 * expression value of TP63) + (-0.057 * expression value of YAP1) + (0.498 * expression value of ZFP64). There were two risk categories for patients: high-risk and low-risk. A cutoff point was determined based on the training set’s median risk score.

**Table 2:** Each genetic parameter of the risk signature.

**mRNA**	**Coefficient**	**HR**	**95%CI**	***P***
EN1	-0.908	0.403	0.178-0.913	0.029
ETV1	0.214	1.239	1.063-1.444	0.006
HDAC2	0.238	1.269	1.152-1.397	0.001
IRF8	-0.058	0.944	0.887-1.004	0.068
MYC	0.017	1.017	1.005-1.030	0.008
SNAI2	0.020	1.020	0.997-1.043	0.094
TAZ	0.030	1.031	1.001-1.061	0.043
TP53	-0.073	0.930	0.879-0.983	0.011
TP63	-0.218	0.804	0.600-1.077	0.143
YAP1	-0.057	0.944	0.908-0.982	0.004
ZFP64	0.498	1.645	1.295-2.091	0.001

**Figure 5:**
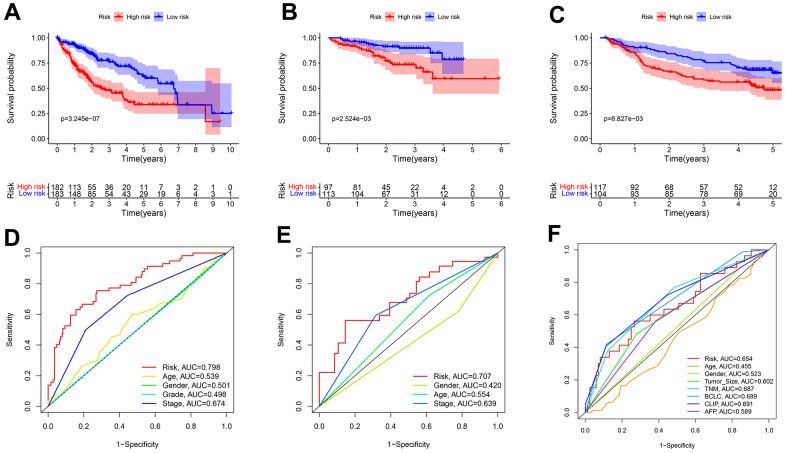
**Predicting patient survival for the transcriptional addiction gene signature.** (**A**) Survival analysis of different risk groups based on 365 patients from TCGA-LIHC set. (**B**) Survival analysis of risk groups based on 210 patients from ICGC-JP set. (**C**) Survival analysis of different risk groups based on 221 patients from GEO-GSE14520 set. (**D**) The time-dependent ROC curve of the risk score and clinicopathological characteristics in TCGA-LIHC set. (**E**) The time-dependent ROC curve of the risk score and clinicopathological characteristics in ICGC-JP set. (**F**) The time-dependent ROC curve of the risk score and clinicopathological characteristics in GEO-GSE14520 set.

**Figure 6:**
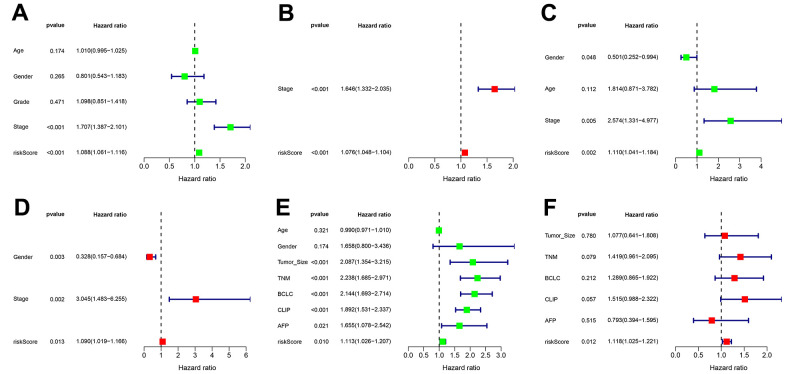
**Independent prognostic value of transcriptional addiction gene signature.** (**A**, **B**) Univariate and multivariate regression analyses for hazard ratio values of risk score and clinical characteristics in TCGA-LIHC set. (**C**, **D**) Univariate and multivariate regression analyses for hazard ratio values of risk score and clinical characters in ICGC-JP set. (**E**, **F**) Univariate and multivariate regression analyses for hazard ratio values of risk score and clinical characters in GEO-GSE14520 set.

In all sets, there was a greater median overall survival time among the patients in the low-risk group compared to those in the high-risk group (*p* < 0.01) (Figure 5A–5C). In the TCGA-LIHC and ICGC-JP cohorts, the area under the curve (AUC) for the risk score was higher than that for the other clinical factors (Figure 5D, 5E). In the GEO cohort, the AUC for the risk score demonstrated slightly lower performance (Figure 5F). The additional GEO verification set (GSE20140) is provided in the Supplementary Figure 1. This difference could be related to the patient’s geographical area, tumor stage, and other factors. The results of the univariate/multivariate Cox regression analyses were consistent across multiple cohorts, and they all supported that risk score was an independent risk predictor of patient survival (HR > 1 and *p* < 0.05) (Figure 6).

### Correlation of risk signature with therapeutic drugs and immune status

**Figure 7:**
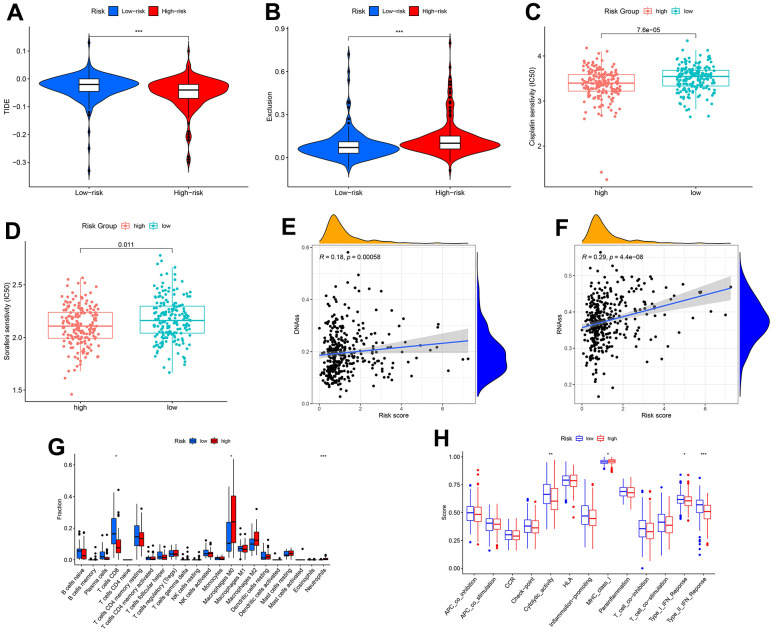
**Correlation of transcriptional addiction gene signature with therapeutic drug sensitivity and immune status in TCGA-LIHC set.** (**A**) TIDE scores in different risk groups. (**B**) Exclusion scores in different risk groups. (**C**) Cisplatin sensitivity in different risk groups. (**D**) Sorafenib sensitivity in different risk groups. (**E**) Correlation between DNA stemness score and risk score. (**F**) Correlation between RNA stemness score and risk score. (**G**) Immune cell contents in different risk groups. (**H**) Immune function scores in different risk groups. *p < 0.05; **p < 0.01; ***p < 0.001.

There were differences in TIDE and exclusion scores between risk groups (*p* < 0.001) (Figure 7A, 7B). Thus, high-risk patients were more likely to benefit from immunotherapy. Similarly, chemotherapy and targeted therapy are more effective in the high-risk group, considering the lower IC50 values in this group (Figure 7C, 7D). Both DNA and RNA stemness scores were positively correlated with the risk score (*p* < 0.001) (Figure 7E, 7F). This result could provide a reference for targeted cancer stem cell therapy. Finally, we identified several immune cell contents and immune function scores associated with risk signature, including T cells CD8, macrophages M0, cytolytic activity, MHC class I, type I IFN response, and type II IFN response (Figure 7G, 7H).

### HDAC2 expression and clinical value in HCC

**Figure 8:**
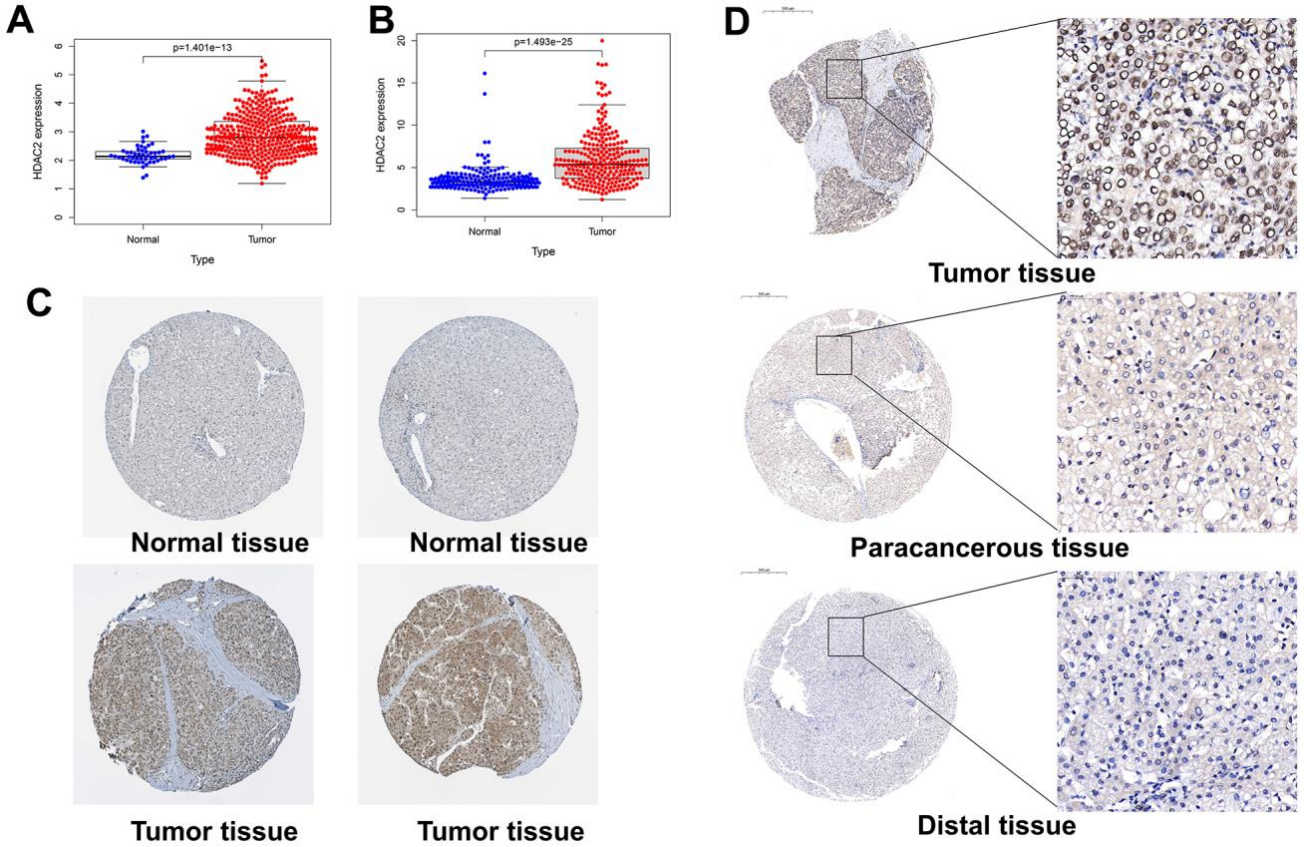
**Expression levels of HDAC2 in different tissues.** (**A**) HDAC2 expression in normal and tumor tissues in the TCGA-LIHC cohort. (**B**) HDAC2 expression in normal and tumor tissues in the ICGC-JP cohort. (**C**) The protein level of HDAC2 in normal and tumor tissues in HPA database. (**D**) The protein levels of HDAC2 in tumor tissue, paracancerous tissue, and distal tissue in the tissue microarray.

By bioinformatics mining, HDAC2 expression was higher in the cancer tissues than that in the adjacent tissues in the TCGA-LIHC and ICGC-JP sets (*p* < 0.001) (Figure 8A, 8B). In the HPA database, immunohistochemistry staining also identified higher levels of HDAC2 protein in HCC tissues compared with those in normal liver tissues (Figure 8C). Tissue microarrays likewise demonstrated high levels of HDAC2 protein in tumor tissues compared with those of paracancerous tissues and distal tissues (Figure 8D).

**Figure 9:**
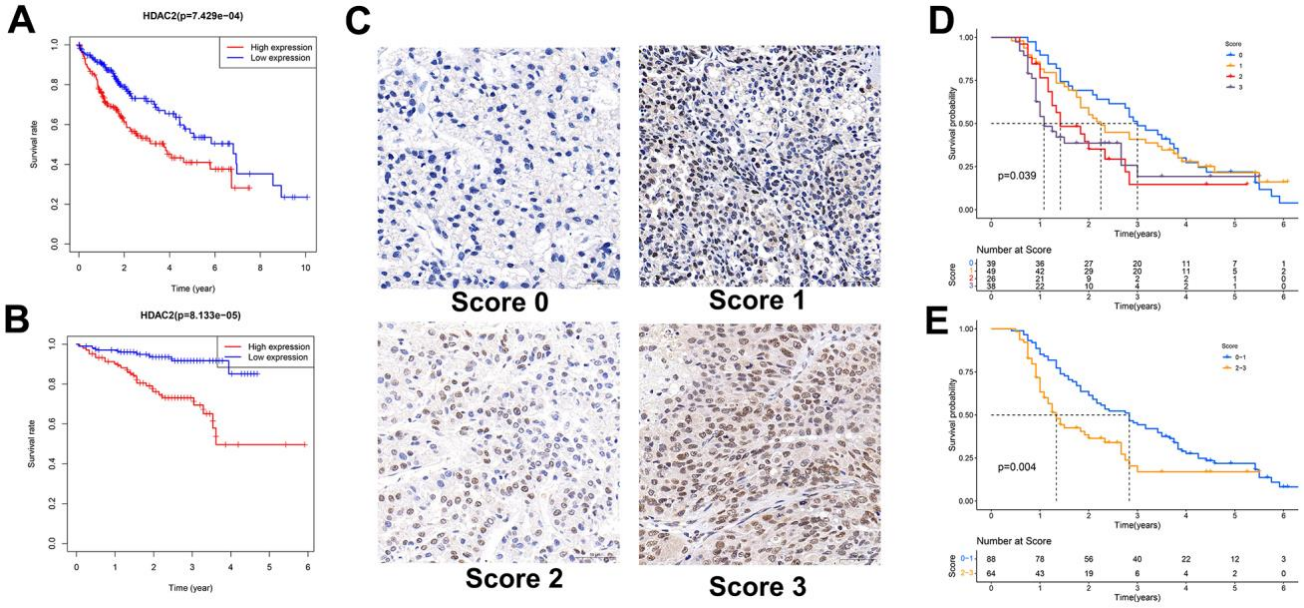
**Relationship between HDAC2 expression and patient survival.** (**A**) Kaplan-Meier survival analysis of patients with different HDAC2 expressions in TCGA-LIHC cohort. (**B**) Kaplan-Meier survival analysis of patients with different HDAC2 expressions in ICGC-JP cohort. (**C**) Scored for each sample of the tissue microarray. (**D**, **E**) Kaplan-Meier survival analysis of patients with different tissue scores in the tissue microarrays.

**Figure 10:**
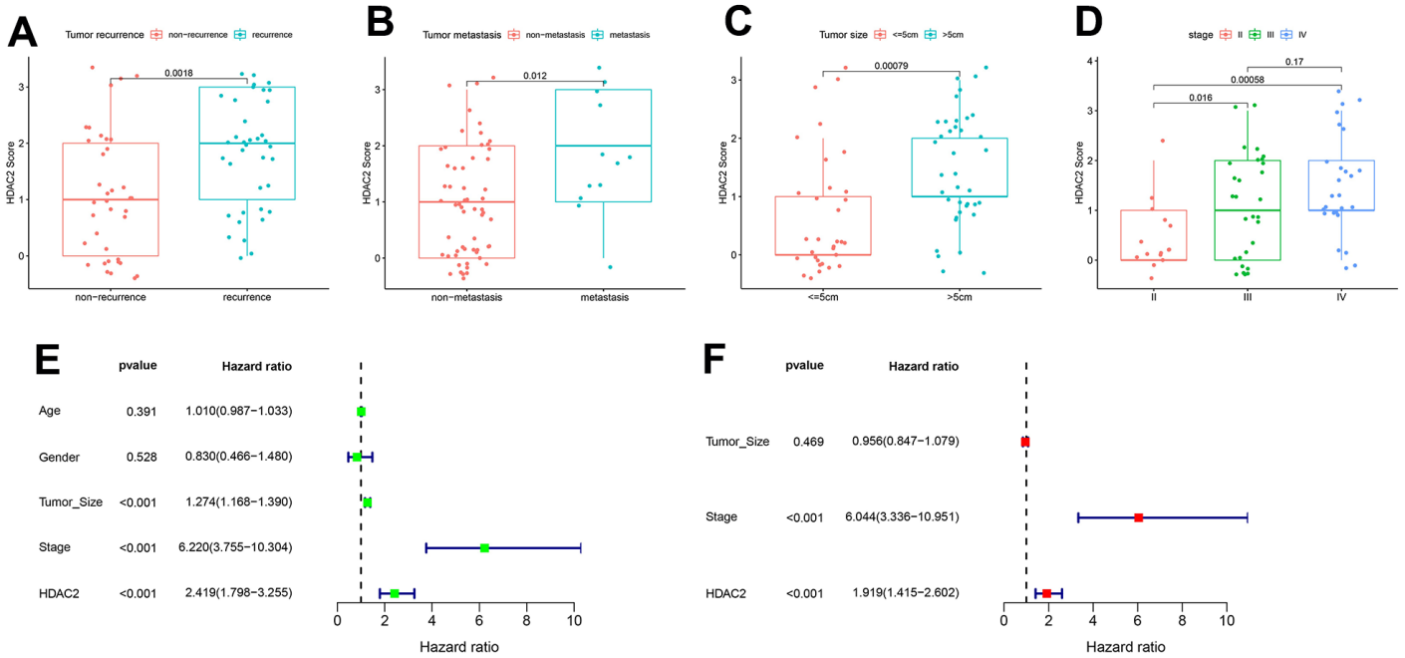
**Correlation of HDAC2 score with HCC pathological characteristics in the tissue microarray set.** (**A**) HDAC2 score in tumor recurrence and non-recurrence groups. (**B**) HDAC2 score in tumor metastatic and non-metastatic groups. (**C**) HDAC2 score in different tumor size groups. (**D**) HDAC2 score in different tumor stage groups. (**E**, **F**) Univariate and multivariate regression analyses for HDAC2 score as an independent prognostic factor.

In the TCGA-LIHC and ICGC-JP sets, the median HDAC2 expression value was used for grouping patients. The patients with high HDAC2 expression had a shorter median overall survival time than low expression patients (*p* < 0.001) (Figure 9A, 9B). The HDCA2 protein level was scored for each sample of the tissue microarrays [40, 41] (Figure 9C). Consistent results were observed across all survival analyses. The patients with higher ICH scores had shorter survival times (Figure 9D, 9E). HDAC2—an oncogene—may affect the survival of HCC patients. Furthermore, HDAC2 was up-regulated in HCC recurrent and metastatic tissues (Figure 10A, 10B). HDAC2 scores also differed in samples with different tumor sizes and stages (Figure 10C, 10D). HDAC2 score could be an independent prognostic factor for HCC patients (Figure 10E, 10F).

## DISCUSSION

The mortality rate for liver cancer is the fourth highest among all cancers [1]. Because of the lack of early-stage symptoms, most patients do not benefit from surgery at the initial diagnosis [4, 42]. The conventional treatment for HCC is ineffective, and many exploratory clinical trials have failed to meet their endpoints [43]. The development of HCC is a complex process involving multiple gene regulatory networks at the molecular level. The conventional early warning markers primarily focus on tumor volume and clinical symptoms, with insufficient sensitivity and specificity. In the precision medicine era, studies on genetic characteristics may provide us with new directions. A growing number of studies have proposed new biomarkers for liver cancer, such as the cancer driver gene [44], autophagy [45], and ferroptosis-related gene signature [46]. The present study identified novel transcriptional addiction gene-related signatures and nomograms. The transcriptional addiction gene signature demonstrated better sensitivity and specificity compared with conventional clinical factors when validated across multiple platforms.

The HCC genome is highly heterogeneous, with transcriptional dysregulation of many genes. However, transcriptional addiction has received little attention in the field of liver cancer. Francesca et al. [7] found that the oncogenic properties of BRD4 were dependent on the YAP/TAZ transcriptional response. BET inhibitors could limit the liver growth caused by YAP transcription. There are also several other transcriptional addiction genes. For example, CDK7 is a famous transcriptional addiction-related gene that affects the progression, metastasis, and prognosis of many cancers [6, 16]. SOX2 transcription is associated with cell growth in lung and esophageal cancers [26, 31]. Transcriptional addiction may be a vital physiological and pathological mechanism in various processes, including tumorigenesis, development, and metastasis.

Our study examined the expression characteristics of transcriptional addiction genes in HCC. We identified several DEGs. The functional role of most genes has not been a concern in HCC. For instance, high expression of DOT1L may be associated with poor prognosis in HCC patients by mining public cancer databases. As a transcriptional addiction gene, DOT1L-mediated transcriptional regulatory mechanisms were involved in developing and maintaining MLL leukemia [13]. Yu et al. [47] reported that TRPS1 could facilitate the invasion and proliferation of HCC cells. Overexpression of TRPS1 was associated with tumor size and stage. However, the specific mechanism and prognostic value of TRPS1 in HCC are still unclear. We compiled a list of the transcriptional addiction-related genes to serve as a reference for subsequent studies.

It is common for patients with the same tumor stage and receiving the same treatment to have different outcomes. Targeting key transcriptional addiction genes may facilitate the development of biomarkers and therapeutic strategies. Wei et al. [48] reported that ZFP64 was up-regulated in tumor tissues of immunotherapy-insensitive HCC patients. Inhibiting PKCα/ZFP64/CSF1 axis could overcome anti-PD1 resistance in HCC. ETV1 may facilitate the invasion and metastasis of HCC by up-regulating metastasis-related genes; PTK2 and c-MET inhibitors could significantly inhibit this process [49]. Our study found that different groups of patients responded differently to cisplatin, sorafenib, and immunotherapy, which could guide drug selection for patients.

In combination with public cancer databases and tissue microarrays, this study investigates the role of HDAC2 in HCC. Aberrant expression and mutations of HDACs in cancer have been widely reported. HDAC10 regulates the stemness characteristics of lung cancer cells [50]. HDAC10 also serves as a new therapeutic target in ovarian and gastric cancers [51, 52]. Some selective HDAC6 inhibitors, such as ricolinostat and ACY-1215, have been applied in clinical trials [53]. As for HDAC2, the related studies mainly focus on colorectal cancer (CRC). Tang et al. [54] found that transcriptional regulation associated with the p300/YY1/miR-500a-5p/HDAC2 signaling axis was a critical step that affects the proliferation of CRC cells. Cui et al. [55] reported that HDAC2 promoted CRC cell proliferation by regulating GLI2 expression. HDAC2 may also facilitate multiple biological behaviors in hepatocellular carcinoma. Jin et al. [56] found that HDAC2 inhibition could enhance HCC radiosensitivity. Nam et al. [57] reported that HDAC2 was involved in HCC progression through feedback control of mTOR and AKT. HDAC2 may also affect the M2 macrophage migration and immune escape in HCC [58]. Our study demonstrated that high expression of HDAC2 could contribute to poor patient outcomes. HDAC2—an oncogene—contributes to metastatic and recurrent HCC.

## CONCLUSIONS

Cancer develops and metastasizes via transcriptional addiction, which is common and critical. As demonstrated in our study, transcriptional addiction genes were implicated in HCC. Transcriptional addiction-related nomograms and signatures could provide significant predictions of survival time for patients. Gene expression characteristics determined the sensitivity of patients to therapeutic agents. HDAC2 was highly expressed in tumor tissues, and its level was associated with patients’ survival. Multiple databases confirmed our findings. Our study identified new biomarkers that can be used to predict survival and select suitable therapeutic drugs for HCC patients.

## MATERIALS AND METHODS

### Data sources

This study’s data were obtained from three publicly available databases and three tissue microarrays. We obtained HCC RNA-seq and clinical information from The Cancer Genome Atlas (TCGA) (https://portal.gdc.cancer.gov/), International Cancer Genome Consortium (ICGC) (https://icgc.org/), and Gene Expression Omnibus (GEO) (https://www.ncbi.nlm.nih.gov/geo/) databases. The TCGA-LIHC set, ICGC-JP set, and GEO-GSE14520 set included 365, 210, and 221 HCC cases, respectively. Specific clinical information is provided in the Supplementary Table 1. The protein levels of transcriptional addiction genes in normal and tumor tissues were analyzed using the Human Protein Atlas (HPA) database. RNA-seq profiles were normalized using the “limma” and “sva” packages in R v4.2.1 software (https://www.r-project.org/).

Tissue microarray 1 (Outdo Biotech, Shanghai) contains tumor tissue, paracancerous tissue, and distal tissue from three HCC patients. The other tissue microarrays (Servicebio, Wuhan) contain tumor tissues from 152 HCC patients and their corresponding survival data. Tissue microarrays were used to verify the correlation between HDAC2 expression and patient survival.

### Differentially expressed genes in different sets

The differentially expressed genes (DEGs) were identified by comparing transcriptional addiction gene expression in tumor and paracancerous tissues. The cutoff was set as *p* < 0.05. We visualized the overlapping genes from different datasets using Venn plots (https://bioinformatics.psb.ugent.be/webtools/Venn/).

### Construction of cancer transcription factor co-expression network and gene function analysis in the TCGA-LIHC set

Cistrome Cancer (http://cistrome.org/) provides a list of cancer transcription factors. We analyzed the correlation between 318 cancer transcription factors and DEGs in the TCGA-LIHC set. The threshold value was *p* < 0.05 and |R^2^| > 0.5. The co-expression network of these genes was visualized using Cytoscape v3.8.1. Gene Ontology (GO) and Kyoto Encyclopedia of Genes and Genomes (KEGG) suggest a range of functional and pathway sets in which DEGs may influence the biological behavior of HCC. This process was performed using the “enrichplot” R package.

### Construction and validation of clustered subgroups and nomogram in the TCGA-LIHC set

Gene clustering was performed using the “ConsensusClusterPlus” package according to the expression of DEGs in the TCGA-LIHC set. The distribution among those cases was mapped using principal component analysis (PCA). Survival analysis verifies whether there were differences in survival rates between subgroups of patients. Further, clinical data and clustered subgroups were integrated to construct a nomogram using the “nomogram” package. The calibration curve and concordance index (C-index) were used to verify the specificity and predictive accuracy of the nomogram. The nomogram combined with clinicopathological characteristics was included for univariate and multivariate regression analyses.

### Construction and validation of transcriptional addiction gene signature

With the TCGA-LIHC set as the training set, we developed a risk signature using multivariate Cox proportional regression. The ICGC-JP and GEO sets were defined as test sets. All cases were divided into high-risk and low-risk groups based on the median risk score of the training set. Survival analysis, receiver operating characteristic (ROC) curve, and univariate/multivariate regression were used to analyze the prognostic value of risk score in HCC patients.

The tumor immune dysfunction and exclusion (TIDE) score (http://tide.dfci.harvard.edu/) reflects the sample’s sensitivity to immunotherapy. We analyzed the sensitivity of different risk samples to immunotherapy, cisplatin, and sorafenib treatment. We evaluated the correlation between risk score and stem cell content by calculating DNA and RNA stemness scores. Finally, we evaluated the immune cell contents and immune function scores of different samples through the CIBERSORT algorithm (https://cibersortx.stanford.edu/).

### Tissue microarrays and immunohistochemistry detection

We extracted the HDAC2 expression profiles of all cases from the TCGA and ICGC datasets and analyzed the distribution of HDAC2 in tumor tissues and paracancerous tissues. Tissue microarrays and the HPA database were used to verify HDAC2 protein levels in various types of tissues. We performed immunohistochemistry (IHC) of HDAC2 on the tissue microarrays. We incubated the tissue microarrays with the primary antibody against HDAC2 (Servicebio, GB11371, Wuhan), followed by the secondary antibody (Servicebio, GB23303, Wuhan) and 3,3’-diaminobenzidine (DAB) IHC kit (DAKO, K5007, Denmark). The intensity of HDAC2 staining was scored as 0, 1, 2, and 3 for no, low, medium, and high staining, respectively. We pooled these data and analyzed the correlation between HDAC2 protein levels and survival time of HCC patients in TCGA, ICGC, and tissue microarray sets. Univariate and multivariate regression analyses were also performed.

### Statistical analysis

Expression or score differences between groups were analyzed using the Wilcoxon rank-sum test. Spearman’s rank correlation coefficient was used to determine the correlation between risk and stemness scores. For survival analysis, the Kaplan-Meier and log-rank tests were used. The independent prognostic value of the nomogram, risk signature, and HDAC2 score was performed with univariate/multivariate regression analyses. All statistical analysis procedures were performed using R v4.2.1 software.

### Availability of data and material

Publicly available cancer databases were obtained from The Cancer Genome Atlas (TCGA) (https://portal.gdc.cancer.gov/) and International Cancer Genome Consortium (ICGC) (https://icgc.org/) databases.

### Consent for publication

All authors have read and approved the content and agree to submit for consideration for publication in the journal.

## Supplementary Material

Supplementary Figure 1

Supplementary Table 1

## References

[1] Sung H, Ferlay J, Siegel RL, Laversanne M, Soerjomataram I, Jemal A, Bray F. Global Cancer Statistics 2020: GLOBOCAN Estimates of Incidence and Mortality Worldwide for 36 Cancers in 185 Countries. CA Cancer J Clin. 2021; 71:209–49. 10.3322/caac.21660 33538338

[2] Chen W, Zheng R, Baade PD, Zhang S, Zeng H, Bray F, Jemal A, Yu XQ, He J. Cancer statistics in China, 2015. CA Cancer J Clin. 2016. 10.3322/caac.21338 26808342

[3] Villanueva A. Hepatocellular Carcinoma. N Engl J Med. 2019; 380:1450–62. 10.1056/NEJMra1713263 30970190

[4] Hepatocellular carcinoma. Nat Rev Dis Primers. 2021; 7:7. 10.1038/s41572-021-00245-6 33479233

[5] Vervoort SJ, Devlin JR, Kwiatkowski N, Teng M, Gray NS, Johnstone RW. Targeting transcription cycles in cancer. Nat Rev Cancer. 2022; 22:5–24. 10.1038/s41568-021-00411-8 34675395

[6] Bradner JE, Hnisz D, Young RA. Transcriptional Addiction in Cancer. Cell. 2017; 168:629–43. 10.1016/j.cell.2016.12.013 28187285 PMC5308559

[7] Zanconato F, Battilana G, Forcato M, Filippi L, Azzolin L, Manfrin A, Quaranta E, Di Biagio D, Sigismondo G, Guzzardo V, Lejeune P, Haendler B, Krijgsveld J, et al. Transcriptional addiction in cancer cells is mediated by YAP/TAZ through BRD4. Nat Med. 2018; 24:1599–610. 10.1038/s41591-018-0158-8 30224758 PMC6181206

[8] Zhang Z, Peng H, Wang X, Yin X, Ma P, Jing Y, Cai MC, Liu J, Zhang M, Zhang S, Shi K, Gao WQ, Di W, Zhuang G. Preclinical Efficacy and Molecular Mechanism of Targeting CDK7-Dependent Transcriptional Addiction in Ovarian Cancer. Mol Cancer Ther. 2017; 16:1739–50. 10.1158/1535-7163.MCT-17-0078 28572168

[9] Harada T, Heshmati Y, Kalfon J, Perez MW, Xavier Ferrucio J, Ewers J, Hubbell Engler B, Kossenkov A, Ellegast JM, Yi JS, Bowker A, Zhu Q, Eagle K, et al. A distinct core regulatory module enforces oncogene expression in KMT2A-rearranged leukemia. Genes Dev. 2022; 36:368–89. 10.1101/gad.349284.121 35301220 PMC8973843

[10] Taylor SJ, Sundaravel S, Steidl U. Exploiting a key transcriptional dependency: ZMYND8 and IRF8 in AML. Mol Cell. 2021; 81:3445–6. 10.1016/j.molcel.2021.08.013 34478652

[11] Cao Z, Budinich KA, Huang H, Ren D, Lu B, Zhang Z, Chen Q, Zhou Y, Huang YH, Alikarami F, Kingsley MC, Lenard AK, Wakabayashi A, et al. ZMYND8-regulated IRF8 transcription axis is an acute myeloid leukemia dependency. Mol Cell. 2021; 81:3604–22.e10. 10.1016/j.molcel.2021.07.018 34358447 PMC8932643

[12] Wang L, Hu C, Wang A, Chen C, Wu J, Jiang Z, Zou F, Yu K, Wu H, Liu J, Wang W, Wang Z, Wang B, et al. Discovery of a novel and highly selective CDK9 kinase inhibitor (JSH-009) with potent antitumor efficacy in preclinical acute myeloid leukemia models. Invest New Drugs. 2020; 38:1272–81. 10.1007/s10637-019-00868-3 31872348

[13] Xiao R, Wang H, Liang K. Transcriptional addiction in mixed lineage leukemia: new avenues for target therapies. Blood Sci. 2019; 1:50–6. 10.1097/BS9.0000000000000011 35402805 PMC8975088

[14] Transcription Factor Motif Density Can Confer Transcriptional Addiction. Cancer Discov. 2019; 9:161. 10.1158/2159-8290.CD-RW2018-21234468357

[15] Lu B, Klingbeil O, Tarumoto Y, Somerville TD, Huang YH, Wei Y, Wai DC, Low JK, Milazzo JP, Wu XS, Cao Z, Yan X, Demerdash OE, et al. A Transcription Factor Addiction in Leukemia Imposed by the MLL Promoter Sequence. Cancer Cell. 2018; 34:970–81.e8. 10.1016/j.ccell.2018.10.015 30503706 PMC6554023

[16] Meng W, Wang J, Wang B, Liu F, Li M, Zhao Y, Zhang C, Li Q, Chen J, Zhang L, Tang Y, Ma J. CDK7 inhibition is a novel therapeutic strategy against GBM both *in vitro* and *in vivo*. Cancer Manag Res. 2018; 10:5747–58. 10.2147/CMAR.S183696 30532595 PMC6245350

[17] Decaesteker B, Denecker G, Van Neste C, Dolman EM, Van Loocke W, Gartlgruber M, Nunes C, De Vloed F, Depuydt P, Verboom K, Rombaut D, Loontiens S, De Wyn J, et al. TBX2 is a neuroblastoma core regulatory circuitry component enhancing MYCN/FOXM1 reactivation of DREAM targets. Nat Commun. 2018; 9:4866. 10.1038/s41467-018-06699-9 30451831 PMC6242972

[18] Rusan M, Li K, Li Y, Christensen CL, Abraham BJ, Kwiatkowski N, Buczkowski KA, Bockorny B, Chen T, Li S, Rhee K, Zhang H, Chen W, et al. Suppression of Adaptive Responses to Targeted Cancer Therapy by Transcriptional Repression. Cancer Discov. 2018; 8:59–73. 10.1158/2159-8290.CD-17-0461 29054992 PMC5819998

[19] Chen Y, Xu L, Lin RY, Müschen M, Koeffler HP. Core transcriptional regulatory circuitries in cancer. Oncogene. 2020; 39:6633–46. 10.1038/s41388-020-01459-w 32943730 PMC7581508

[20] Chen Y, Xu L, Mayakonda A, Huang ML, Kanojia D, Tan TZ, Dakle P, Lin RY, Ke XY, Said JW, Chen J, Gery S, Ding LW, et al. Bromodomain and extraterminal proteins foster the core transcriptional regulatory programs and confer vulnerability in liposarcoma. Nat Commun. 2019; 10:1353. 10.1038/s41467-019-09257-z 30903020 PMC6430783

[21] Yuan J, Li X, Yu S. CDK7-dependent transcriptional addiction in bone and soft tissue sarcomas: Present and Future. Biochim Biophys Acta Rev Cancer. 2022; 1877:188680. 10.1016/j.bbcan.2022.188680 35051528

[22] Ran L, Chen Y, Sher J, Wong EW, Murphy D, Zhang JQ, Li D, Deniz K, Sirota I, Cao Z, Wang S, Guan Y, Shukla S, et al. FOXF1 Defines the Core-Regulatory Circuitry in Gastrointestinal Stromal Tumor. Cancer Discov. 2018; 8:234–51. 10.1158/2159-8290.CD-17-0468 29162563 PMC5809271

[23] Vitale E, Sauta E, Gugnoni M, Torricelli F, Manicardi V, Ciarrocchi A. A multimodal integrative approach to model transcriptional addiction of thyroid cancer on RUNX2. Cancer Commun (Lond). 2022; 42:892–6. 10.1002/cac2.12292 35451571 PMC9456690

[24] Ray T, Ryusaki T, Ray PS. Therapeutically Targeting Cancers That Overexpress FOXC1: A Transcriptional Driver of Cell Plasticity, Partial EMT, and Cancer Metastasis. Front Oncol. 2021; 11:721959. 10.3389/fonc.2021.721959 34540690 PMC8446626

[25] Cao X, Dang L, Zheng X, Lu Y, Lu Y, Ji R, Zhang T, Ruan X, Zhi J, Hou X, Yi X, Li MJ, Gu T, et al. Targeting Super-Enhancer-Driven Oncogenic Transcription by CDK7 Inhibition in Anaplastic Thyroid Carcinoma. Thyroid. 2019; 29:809–23. 10.1089/thy.2018.0550 30924726

[26] Jiang YY, Jiang Y, Li CQ, Zhang Y, Dakle P, Kaur H, Deng JW, Lin RY, Han L, Xie JJ, Yan Y, Doan N, Zheng Y, et al. TP63, SOX2, and KLF5 Establish a Core Regulatory Circuitry That Controls Epigenetic and Transcription Patterns in Esophageal Squamous Cell Carcinoma Cell Lines. Gastroenterology. 2020; 159:1311–27.e19. 10.1053/j.gastro.2020.06.050 32619460

[27] Li CL, Huang CW, Ko CJ, Fang SY, Ou-Yang FU, Pan MR, Luo CW, Hou MF. Curcumol Suppresses Triple-negative Breast Cancer Metastasis by Attenuating Anoikis Resistance via Inhibition of Skp2-mediated Transcriptional Addiction. Anticancer Res. 2020; 40:5529–38. 10.21873/anticanres.14565 32988876

[28] Peluffo G, Subedee A, Harper NW, Kingston N, Jovanović B, Flores F, Stevens LE, Beca F, Trinh A, Chilamakuri CSR, Papachristou EK, Murphy K, Su Y, et al. *EN1* Is a Transcriptional Dependency in Triple-Negative Breast Cancer Associated with Brain Metastasis. Cancer Res. 2019; 79:4173–83. 10.1158/0008-5472.CAN-18-3264 31239270 PMC6698222

[29] Witwicki RM, Ekram MB, Qiu X, Janiszewska M, Shu S, Kwon M, Trinh A, Frias E, Ramadan N, Hoffman G, Yu K, Xie Y, McAllister G, et al. TRPS1 Is a Lineage-Specific Transcriptional Dependency in Breast Cancer. Cell Rep. 2018; 25:1255–67.e5. 10.1016/j.celrep.2018.10.023 30380416 PMC6366939

[30] Wang Y, Zhang T, Kwiatkowski N, Abraham BJ, Lee TI, Xie S, Yuzugullu H, Von T, Li H, Lin Z, Stover DG, Lim E, Wang ZC, et al. CDK7-dependent transcriptional addiction in triple-negative breast cancer. Cell. 2015; 163:174–86. 10.1016/j.cell.2015.08.063 26406377 PMC4583659

[31] Hur JY, Kim HR, Lee JY, Park S, Hwang JA, Kim WS, Yoon S, Choi CM, Rho JK, Lee JC. CDK7 inhibition as a promising therapeutic strategy for lung squamous cell carcinomas with a SOX2 amplification. Cell Oncol (Dordr). 2019; 42:449–58. 10.1007/s13402-019-00434-2 30838525 PMC12994335

[32] Liang K, Smith ER, Aoi Y, Stoltz KL, Katagi H, Woodfin AR, Rendleman EJ, Marshall SA, Murray DC, Wang L, Ozark PA, Mishra RK, Hashizume R, et al. Targeting Processive Transcription Elongation via SEC Disruption for MYC-Induced Cancer Therapy. Cell. 2018; 175:766–79.e17. 10.1016/j.cell.2018.09.027 30340042 PMC6422358

[33] Augert A, MacPherson D. Treating transcriptional addiction in small cell lung cancer. Cancer Cell. 2014; 26:783–4. 10.1016/j.ccell.2014.11.012 25490443

[34] Huang CS, Xu QC, Dai C, Wang L, Tien YC, Li F, Su Q, Huang XT, Wu J, Zhao W, Yin XY. Nanomaterial-Facilitated Cyclin-Dependent Kinase 7 Inhibition Suppresses Gallbladder Cancer Progression via Targeting Transcriptional Addiction. ACS Nano. 2021; 15:14744–55. 10.1021/acsnano.1c04570 34405985

[35] Lu P, Geng J, Zhang L, Wang Y, Niu N, Fang Y, Liu F, Shi J, Zhang ZG, Sun YW, Wang LW, Tang Y, Xue J. THZ1 reveals CDK7-dependent transcriptional addictions in pancreatic cancer. Oncogene. 2019; 38:3932–45. 10.1038/s41388-019-0701-1 30692639

[36] Kalan S, Amat R, Schachter MM, Kwiatkowski N, Abraham BJ, Liang Y, Zhang T, Olson CM, Larochelle S, Young RA, Gray NS, Fisher RP. Activation of the p53 Transcriptional Program Sensitizes Cancer Cells to Cdk7 Inhibitors. Cell Rep. 2017; 21:467–81. 10.1016/j.celrep.2017.09.056 29020632 PMC5687273

[37] Yang Y, Jiang D, Zhou Z, Xiong H, Yang X, Peng G, Xia W, Wang S, Lei H, Zhao J, Qian Z, Wu S, Pang J. CDK7 blockade suppresses super-enhancer-associated oncogenes in bladder cancer. Cell Oncol (Dordr). 2021; 44:871–87. 10.1007/s13402-021-00608-x 33905040 PMC12980791

[38] Constantin TA, Greenland KK, Varela-Carver A, Bevan CL. Transcription associated cyclin-dependent kinases as therapeutic targets for prostate cancer. Oncogene. 2022; 41:3303–15. 10.1038/s41388-022-02347-1 35568739 PMC9187515

[39] Rasool RU, Natesan R, Deng Q, Aras S, Lal P, Sander Effron S, Mitchell-Velasquez E, Posimo JM, Carskadon S, Baca SC, Pomerantz MM, Siddiqui J, Schwartz LE, et al. CDK7 Inhibition Suppresses Castration-Resistant Prostate Cancer through MED1 Inactivation. Cancer Discov. 2019; 9:1538–55. 10.1158/2159-8290.CD-19-0189 31466944 PMC7202356

[40] Ler SY, Leung CH, Khin LW, Lu GD, Salto-Tellez M, Hartman M, Iau PT, Yap CT, Hooi SC. HDAC1 and HDAC2 independently predict mortality in hepatocellular carcinoma by a competing risk regression model in a Southeast Asian population. Oncol Rep. 2015; 34:2238–50. 10.3892/or.2015.4263 26352599 PMC4583520

[41] Luo Y, Han S, Yan B, Ji H, Zhao L, Gladkich J, Herr I. UHMK1 Is a Novel Marker for Personalized Prediction of Pancreatic Cancer Prognosis. Front Oncol. 2022; 12:834647. 10.3389/fonc.2022.834647 35359403 PMC8960145

[42] Qu C, Wang Y, Wang P, Chen K, Wang M, Zeng H, Lu J, Song Q, Diplas BH, Tan D, Fan C, Guo Q, Zhu Z, et al. Detection of early-stage hepatocellular carcinoma in asymptomatic HBsAg-seropositive individuals by liquid biopsy. Proc Natl Acad Sci USA. 2019; 116:6308–12. 10.1073/pnas.1819799116 30858324 PMC6442629

[43] Kudo M, Arizumi T. Transarterial Chemoembolization in Combination with a Molecular Targeted Agent: Lessons Learned from Negative Trials (Post-TACE, BRISK-TA, SPACE, ORIENTAL, and TACE-2). Oncology. 2017 (Suppl 1); 93:127–34. 10.1159/000481243 29258086

[44] Li G, Du X, Wu X, Wu S, Zhang Y, Xu J, Wang H, Chen T. Large-Scale Transcriptome Analysis Identified a Novel Cancer Driver Genes Signature for Predicting the Prognostic of Patients With Hepatocellular Carcinoma. Front Pharmacol. 2021; 12:638622. 10.3389/fphar.2021.638622 34335239 PMC8322950

[45] Fang Q, Chen H. Development of a Novel Autophagy-Related Prognostic Signature and Nomogram for Hepatocellular Carcinoma. Front Oncol. 2020; 10:591356. 10.3389/fonc.2020.591356 33392087 PMC7775646

[46] Xu Z, Peng B, Liang Q, Chen X, Cai Y, Zeng S, Gao K, Wang X, Yi Q, Gong Z, Yan Y. Construction of a Ferroptosis-Related Nine-lncRNA Signature for Predicting Prognosis and Immune Response in Hepatocellular Carcinoma. Front Immunol. 2021; 12:719175. 10.3389/fimmu.2021.719175 34603293 PMC8484522

[47] Yu SP, Yan RH, Gao J, Song D. Significance of TRPS1 in the development and clinicopathologic of hepatocellular carcinoma. Eur Rev Med Pharmacol Sci. 2020; 24:9325–32. 10.26355/eurrev_202009_23014 33015773

[48] Wei CY, Zhu MX, Zhang PF, Huang XY, Wan JK, Yao XZ, Hu ZT, Chai XQ, Peng R, Yang X, Gao C, Gao J, Wang SW, et al. PKCα/ZFP64/CSF1 axis resets the tumor microenvironment and fuels anti-PD1 resistance in hepatocellular carcinoma. J Hepatol. 2022; 77:163–76. 10.1016/j.jhep.2022.02.019 35219791

[49] Zhang T, Wang Y, Xie M, Ji X, Luo X, Chen X, Zhang B, Liu D, Feng Y, Sun M, Huang W, Xia L. HGF-mediated elevation of ETV1 facilitates hepatocellular carcinoma metastasis through upregulating PTK2 and c-MET. J Exp Clin Cancer Res. 2022; 41:275. 10.1186/s13046-022-02475-2 36109787 PMC9479266

[50] Li Y, Zhang X, Zhu S, Dejene EA, Peng W, Sepulveda A, Seto E. HDAC10 Regulates Cancer Stem-Like Cell Properties in KRAS-Driven Lung Adenocarcinoma. Cancer Res. 2020; 80:3265–78. 10.1158/0008-5472.CAN-19-3613 32540961 PMC7442594

[51] Islam MM, Banerjee T, Packard CZ, Kotian S, Selvendiran K, Cohn DE, Parvin JD. HDAC10 as a potential therapeutic target in ovarian cancer. Gynecol Oncol. 2017; 144:613–20. 10.1016/j.ygyno.2017.01.009 28073598 PMC6020686

[52] Jin Z, Jiang W, Jiao F, Guo Z, Hu H, Wang L, Wang L. Decreased expression of histone deacetylase 10 predicts poor prognosis of gastric cancer patients. Int J Clin Exp Pathol. 2014; 7:5872–9. 25337229 PMC4203200

[53] Li T, Zhang C, Hassan S, Liu X, Song F, Chen K, Zhang W, Yang J. Histone deacetylase 6 in cancer. J Hematol Oncol. 2018; 11:111. 10.1186/s13045-018-0654-9 30176876 PMC6122547

[54] Tang W, Zhou W, Xiang L, Wu X, Zhang P, Wang J, Liu G, Zhang W, Peng Y, Huang X, Cai J, Bai Y, Bai L, et al. The p300/YY1/miR-500a-5p/HDAC2 signalling axis regulates cell proliferation in human colorectal cancer. Nat Commun. 2019; 10:663. 10.1038/s41467-018-08225-3 30737378 PMC6368584

[55] Cui H, Yu W, Yu M, Luo Y, Yang M, Cong R, Chu X, Gao G, Zhong M. GPR126 regulates colorectal cancer cell proliferation by mediating HDAC2 and GLI2 expression. Cancer Sci. 2021; 112:1798–810. 10.1111/cas.14868 33629464 PMC8088945

[56] Jin Q, Hu H, Yan S, Jin L, Pan Y, Li X, Peng Y, Cao P. lncRNA MIR22HG-Derived miR-22-5p Enhances the Radiosensitivity of Hepatocellular Carcinoma by Increasing Histone Acetylation Through the Inhibition of HDAC2 Activity. Front Oncol. 2021; 11:572585. 10.3389/fonc.2021.572585 33718133 PMC7943860

[57] Noh JH, Bae HJ, Eun JW, Shen Q, Park SJ, Kim HS, Nam B, Shin WC, Lee EK, Lee K, Jang JJ, Park WS, Lee JY, Nam SW. HDAC2 provides a critical support to malignant progression of hepatocellular carcinoma through feedback control of mTORC1 and AKT. Cancer Res. 2014; 74:1728–38. 10.1158/0008-5472.CAN-13-2109 24448241

[58] Chen F, Yang L, Peng X, Mao M, Liu X, Song J, Hu J. Histone deacetylase 2 regulates STAT1-dependent upregulation of atypical chemokine receptor 3 to induce M2 macrophage migration and immune escape in hepatocellular carcinoma. Mol Immunol. 2022; 151:204–17. 10.1016/j.molimm.2022.09.005 36179603

