# Supplementary material for: Profiling and integrated analysis of transcriptional addiction gene expression and prognostic value in hepatocellular carcinoma
Source: Aging (Albany NY). 2023 Apr 22;15(8):3141–57. doi: 10.18632/aging.204676 (PMC10188332; doi:10.18632/aging.204676)
Supplement: Supplementary Figure 1 [file aging-15-204676-s001.pdf]

SUPPLEMENTARY FIGURE

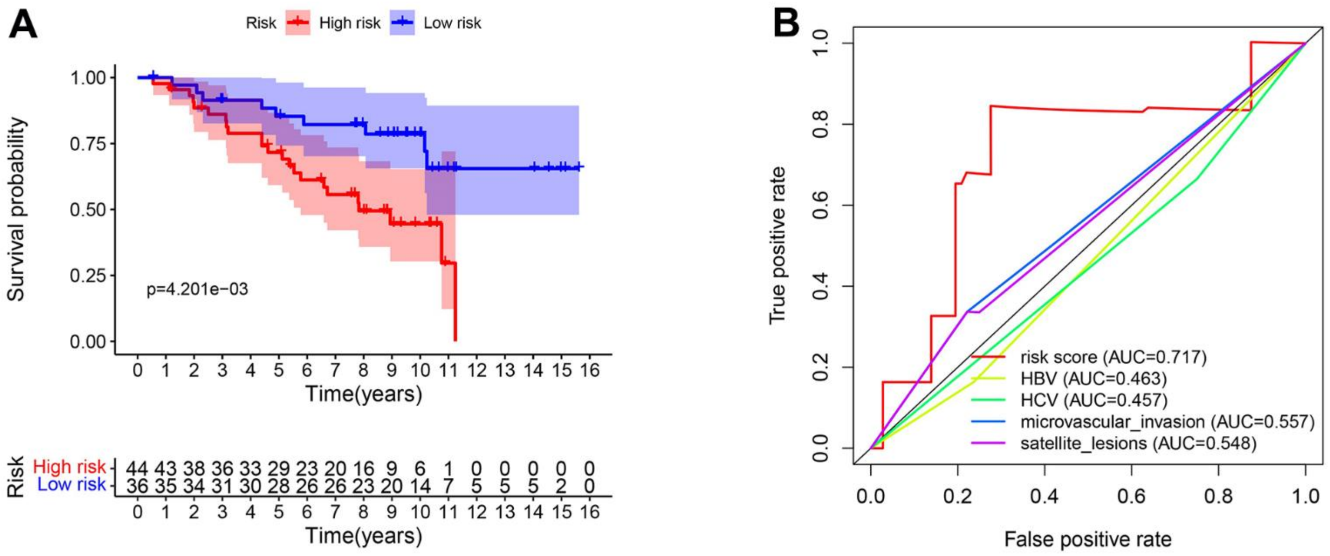

Supplementary Figure 1. Predicting patient survival for the transcriptional addiction gene signature in GEO-GSE20140 set. (**A**) Survival analysis of risk groups based on 80 patients. (**B**) The time-dependent ROC curve of the risk score and clinicopathological characteristics.
